# Adherence to a Mediterranean-style diet and severity of menopausal symptoms in perimenopausal and menopausal women from Australia: a cross-sectional analysis

**DOI:** 10.1007/s00394-024-03462-3

**Published:** 2024-07-18

**Authors:** Maitilde Byrne-Kirk, Evangeline Mantzioris, Nicole Scannell, Anthony Villani

**Affiliations:** 1 School of Health, University of the Sunshine Coast, Sippy Downs, QLD Australia; 2 Clinical and Health Sciences & Alliance for Research in Exercise, Nutrition and Activity (ARENA), University of South Australia, Adelaide, SA Australia

**Keywords:** Mediterranean-style diet, Menopause, Menopausal symptoms, Health Related Quality of Life, Women’s Health

## Abstract

**Purpose:**

To explore the independent associations between adherence to a Mediterranean-style diet and severity of menopausal symptoms in perimenopausal and menopausal women living in Australia.

**Methods:**

Dietary adherence was assessed using the Mediterranean Diet Adherence Screener (MEDAS), the Menopause Rating Scale (MRS) was used to assess the severity of menopausal symptoms, and the 36-item short form survey instrument (SF-36) was used to assess health-related quality of life (HRQoL).

**Results:**

A total of *n* = 207 participants (50.7 ± 4.3 years; BMI: 28.0 ± 7.4 kg/m2) were included in the final analyses. Participants reported low-moderate adherence to a Mediterranean-style diet (5.2 ± 1.8; range: 1–11). Adherence was not associated with severity of menopausal symptoms. However, low consumption of sugar-sweetened beverages was inversely associated with joint and muscle complaints, independent of all covariates (β = -0.149; CI: -0.118, -0.022; *P* = 0.042). Adherence to a Mediterranean-style diet was positively associated with the physical function subscale of HRQoL (β = 0.173, CI: 0.001, 0.029; *P* = 0.031) and a low intake of red and processed meat was positively associated with the general health subscale (β = 0.296, CI: 0.005, 0.014; *P* = < 0.001).

**Conclusion:**

Diet quality may be related to severity of menopausal symptoms and HRQoL in perimenopausal and menopausal women. However, exploration of these findings using longitudinal analyses and robust clinical trials are needed to better elucidate these findings.

## Introduction

The female adult lifecycle can be divided into three distinct phases including premenopausal (e.g., reproductive), perimenopausal (e.g., menopausal transition), and menopausal (e.g., post-menopausal) [1]. Specifically, menopause is an inevitable stage of ageing for women, resulting in the loss of ovarian reproductive function and a permanent cessation of menstruation due to a loss of ovarian follicular activity and a depletion of the female sex hormones, estrogen and progesterone [2, 3]. The median age of onset has previously been reported between 48 and 52 years, with an average duration of 4 years [2]. Nevertheless, the onset of menopause and length of duration varies markedly between individuals. Clinically however, menopause is diagnosed after 12 months of amenorrhoea [3]. Perimenopause, which is also called the menopausal transition, is a period of time during which middle-aged women experience irregular menstruation and intermittent amenorrhea as they progress toward menopause [4]. A number of biological changes occur during the menopause transition including: a disruption of the normal menstrual cycle, a reduction in the number of oocytes, an increase in gonadotrophin levels, particularly follicle stimulating hormones (FSH), and a decline in estrogen levels [5]. During the menopausal transition, women often encounter a range of physical symptoms, including hot flushes, night sweats, mood swings, joint discomfort, sleep disturbances, decreased libido, headache/migraine, cardiometabolic disturbances, weight gain, and loss of bone mineral density [2, 5, 6], all of which can cause distress to women and negatively impact on health-related quality of life (HRQoL) [2, 6, 7].

Given that women will spend approximately one-third of their lives post-menopause, the menopausal transition presents as an important opportunity to initiate lifestyle strategies to help mitigate the severity of symptoms associated with perimenopause and prevent or delay the physical and cardiometabolic consequences associated with post-menopause. In addition to lifestyle factors such as maintaining a healthy body weight, moderating alcohol intake, cessation of smoking and regular physical activity [8], diet and overall nutritional status has been identified as a potentially modifiable factor associated with mitigating the symptoms related to perimenopause or menopause. Evidence from an Australian perspective cohort study showed that adherence to a Mediterranean-style dietary pattern was associated with a decreased risk of vasomotor menopausal symptoms (e.g., hot flushes and night sweats). In contrast, a western-style dietary pattern was associated with an increased frequency and greater intensity of vasomotor symptoms [9]. Similarly, in a cross-sectional analysis of *n* = 400 menopausal women from Iran, Abshirini et al. [10] reported that the total antioxidant capacity of the diet was inversely associated with somatic and psychological symptoms of menopause. A Mediterranean diet (MedDiet), which is often described as an anti-inflammatory dietary pattern, is characterised by a high consumption of fruits, vegetables, legumes, wholegrains and liberal use of extra-virgin olive oil (EVOO); moderate consumption of fermented dairy, eggs and red wine (consumed with meals only); and a low and/or infrequent consumption of red meat and meat products, butter, vegetable oils and processed foods [10]. An increasing number of studies have shown that adherence to a MedDiet is inversely associated with the risk of several chronic diseases including the metabolic syndrome, diabetes, cardiovascular disease, and mental health disorders [11–13]. Although the evidence for this inverse relationship in perimenopausal and menopausal women is scant, there is now a body of evidence suggesting that menopausal symptoms, in particular vasomotor symptoms, are considered precursors or biomarkers for cardiometabolic disease [14]. Being predominately plant-based, the MedDiet is naturally low in saturated fat, and rich in several functional properties, including vitamins and minerals, carotenoids, unsaturated fatty acids, and phenolic compounds, depicted by antioxidant and anti-inflammatory properties [15]. As such, the potential mechanistic benefits of adherence to a MedDiet and attenuation of menopausal symptoms may relate to the anti-inflammatory potential of the dietary pattern, reductions in oxidative stress and a higher intake of antioxidants and phytonutrients. Irrespective, evidence evaluating the transferability and acceptability of a Mediterranean-style diet in non-Mediterranean peri- and post-menopausal women is limited.

This cross-sectional study investigated the relationship between adherence to a Mediterranean-style diet and menopausal symptoms in perimenopausal and menopausal women living in Australia. As a secondary aim, we also explored the association between adherence to a Mediterranean-style diet and HRQoL subscales in this same cohort of women. We also explored whether individual dietary components of a Mediterranean-style diet (as defined by the Mediterranean Diet Adherence Screener (MEDAS)) were independently associated with menopausal symptoms and HRQoL.

## Methods

### Study design, setting and participants

We conducted a cross-sectional online study in Australian perimenopausal or menopausal women aged between 40 and 60 years, and who met (self-reported) at least one of the following Stages of Reproductive Aging Workshop (STRAW) criteria [1]: persistent difference in length of consecutive menstrual cycles (≥ 7 days apart), presence of vasomotor symptoms (e.g., hot flushes and / or night sweats) (perimenopause) or an interval of at least 12 months since the last menstrual period (menopause). Participants who had a previous medical diagnosis of menopause (and met the age criteria) were also eligible to participate. Participants who did not meet the aforementioned criteria or were unable to complete the online survey in English and/or did not permanently reside in Australia were excluded from participation. Participants were recruited either via social media platforms including Facebook, Twitter, Instagram, and LinkedIn; engagement with perimenopausal and menopausal support groups; and networking with local government councils from March 2023 to August 2023. The investigators shared the survey link intermittently on the aforementioned social media platforms across these dates. The survey was constructed and distributed using Qualtrics™ survey software. A link to the survey was disseminated via social media platforms where the study protocol and potential risks were clearly outlined to all interested participants. This study was conducted according to the guidelines described in the Declaration of Helsinki and was approved by the Research Ethics Committees at the University of the Sunshine Coast (S231800) and the University of South Australia (205,348). Prior to participating in the study, participants acknowledged and agreed to an informed consent statement. Lastly, the present study has been reported in accordance with the STROBE-nut checklist [16].

### Data collection

An 86-item self-administered online questionnaire was used to explore the independent relationship between adherence to a Mediterranean-style diet, severity of menopausal symptoms and HRQoL. The questionnaire was divided into four separate sections and included previously validated tools including the MEDAS [17], Menopause Rating Scale (MRS) [18, 19] and the 36-item short form survey instrument (SF-36) [20, 21]. The questionnaire also consisted of open and closed-ended questions relating to participant demographic characteristics which included self-reported height and weight, ethnicity, health-related conditions, medications, physical activity status, sleep duration and smoking status. The questionnaire was designed to be completed in approximately 30 min, however there was no time restrictions applied for completion. The link to the survey was recognizable once only to the server it was sent thus preventing duplication when responding to the survey. The investigators also screened all participant responses (IP address and postcode viewed) to ensure all responses were consistent with the eligibility criteria.

### Mediterranean diet adherence

Adherence to a Mediterranean-style diet was assessed using the validated 14-item MEDAS tool, which was developed and used in the Prevención con Dieta Mediterránea (PREDIMED) study [17]. This tool comprises of twelve questions designed to assess principal dietary constituents of a typical MedDiet and two questions related to food intake behaviours consistent with a MedDiet. Each of the 14 questions was scored dichotomously as 0 or 1, generating a maximum score of 14 where higher scores were reflective of greater adherence to a MedDiet. A score ≥ 10 was suggestive of high adherence, whereas a score ≤ 5 indicates low adherence [17]. For dietary elements consistent with a traditional MedDiet, one point was awarded if participants met the serve size and frequency of consumption criteria for each of the following components:

- 4 or more tablespoons (1 tablespoon = ~ 15 g) of olive oil per day (including use in frying, salads, meals consumed away from the home etc.);
- 2 or more servings of vegetables per day (1 x serve equates to 2 x cups vegetables);
- 3 or more pieces of fruit per day (inclusive of whole, tinned or dried fruit but excludes fruit juice);
- Fewer than 1 serving of red meat or sausages per day (1 x serve equates to 100–150 g);
- Fewer than 1 serving of butter, margarine or cream per day (1 x serve equates to 10 g);
- Fewer than 1 cup (250 ml) of sugar-sweetened beverages per day;
- 7 or more servings of red wine per week (1 x serve equates to 100 ml);
- 3 or more servings of legumes per week (1 x serve equates to 1 x cup);
- 3 or more servings of fish or seafood per week (1 x serve equates to 100–150 g).
- Fewer than 3 commercial sweets or pastries (not homemade) such as cakes, cookies and biscuits per week;
- 3 or more servings of nuts (including peanuts) per week (1 x serve equates to 30 g or approximately 1 x handful);
- 2 or more servings per week of a dish (e.g., pasta / rice / vegetables) made with a traditional sauce including tomatoes, garlic, onion, or leeks sautéed in olive oil.

### Menopausal symptoms

Menopausal symptoms were assessed using the MRS which is a health-related quality of life scale including 11 questions designed to assess the severity of menopausal symptoms and their impact on quality of life [21]. The rating scale was developed due to the lack of standardized scales used to assess the impact and severity of menopausal symptoms on a women’s quality of life [21]. Questions were divided into three subgroups; 1: Somatic symptoms: hot flushes, sweating, trouble sleeping, heart discomfort, joint and muscle complaints; 2: Psychological symptoms: depressive mood, irritability, anxiety, exhaustion, and difficulty concentrating; 3: Urinary-genital symptoms: libido, bladder complaints, and vaginal dryness. Participants were asked to report whether they had experienced any of these symptoms in the previous month. Each symptom was rated on a scale ranging from 0 (no symptom) to 4 (1: mild; 2: moderate; 3: severe; 4: extremely severe). The overall MRS score was calculated by summing the scores from each individual symptom. A score of ≥ 17 was considered a high menopausal symptom score [18, 19].

### Health-related quality of life

HRQoL was assessed using an Australian adapted version of the SF-36 [20, 21]. The survey instrument is a self-administered 36-item validated tool designed to provide aggregated physical and mental component scores based on eight subscales associated with HRQoL, including: Physical Functioning, Role Limitations Due to Physical Health, Role Limitations due to Emotional Health, Bodily Pain, General Health, Vitality, Social Functioning and Mental Health. Scoring values for each subscale ranged from 0 to 100, with higher scores reflective of a higher perceived HRQoL.

### Statistical analysis

All continuous variables are presented as means ± standard deviation (SD), or median and interquartile ranges (IQR), and categorical data presented as frequencies and percentages. Multiple regression diagnostics were performed prior to regression analyses to ensure assumptions of multicollinearity and homoscedasticity were not violated. Multivariable linear regression analysis (and 95% CI) was used to investigate the independent association between adherence to a Mediterranean-style diet, severity of menopausal symptoms and HRQoL subscales using one unadjusted and five adjusted models. We also applied univariable and multivariable regression analysis on the individual dietary constituents of a Mediterranean-style diet to explore their independent relationship with severity of menopausal symptoms and HRQoL. Covariates included in our predictor models included age, BMI, smoking status, use of menopausal (e.g., any form of menopausal hormone therapy) and mood medications and physical activity status. Standardized beta-coefficients were used in the univariable and multivariable linear regressions with z-scores for all outcome variables calculated before running each of the regression models to ensure comparisons across each of the outcomes. Analyses were performed using Statistical Package for the Social Sciences (SPSS) for Windows 27.0 software (IDM Corp., Armonk, NY, USA), with statistical significance set a *P* < 0.05.

## Results

A total of *n* = 229 women participated in the online questionnaire, of which *n* = 207 participants (50.7 ± 4.3 years; BMI: 28.0 ± 7.4 kg/m2) met the inclusion criteria and completed all components of the questionnaire. The majority of participants were born in Australia (*n* = 176; 85%) however a variety of cultural backgrounds were reported with the two most prominent being English (*n* = 118; 57%) and Australian (*n* = 38; 18.3%). A further *n* = 4 (2%) participants identified as First Nation (Indigenous) Australians. Demographic characteristics of the participants are presented in Table 1. Over half of the participants self-reported a diagnosis of menopause (*n* = 123; 60%) and over one-third (*n* = 82; 39.8%) were prescribed menopausal hormone therapy (of any form) for the management of menopausal symptoms. A further one in five women were prescribed with mood-related medications. According to MRS scores, the total sample reported a high severity of menopausal symptoms (17.1 ± 7.2; range: 0–44). Individual symptoms were evenly reported across somatic, psychological and urinary-genital symptoms, with at least one in three women reporting mild-moderate severity for hot flushes, heart discomfort, trouble sleeping, depressive mood, anxiety, irritability, exhaustion, bladder complaints and vaginal dryness. In contrast, scores for HRQoL subscales varied among participants, with vitality being the lowest reported subscale (35.0 (35.0)), and physical functioning being the highest (80.0 (30.0)).

**Table 1:** Participant demographic characteristics

Characteristics	
**Age** (years)	50.7 ± 4.3
**BMI** (kg/m2)	28.0 ± 7.4
**Education status*n***, **%**	
Secondary School	24 (11.7)
Trade/technical/vocational training	24 (11.7)
Diploma	29 (14.1)
Advanced diploma/associate degree	16 (7.8)
Bachelor’s degree	51 (24.8)
Postgraduate degree or doctorate	62 (30.1)
**Menopause Diagnoses*n***, **%**	123 (60)
**Use of menopause medications*n***, **%**	82 (39.8)
**Use of mood medications*n***, **%**	40 (19.4)
**Severity of menopausal symptoms (MRS score)**	17.1 ± 7.2
**MedDiet adherence (MEDAS)**	5.2 ± 1.8
**SF-36 subscales**	
Physical functioning**	80.0 30.0)
Physical health**	37.5 (75.0)
Vitality**	35.0 (35.0)
Emotional health**	66.7 (100.0)
Emotional wellbeing**	60.0 (28.0)
Social function**	62.5 (38.0)
Pain**	67.5 (35.0)
General health**	55.0 (30.0)
**Smoking status*n***,*** %***	
Non-smoker	130 (63.1)
Former smoker	60 (29.1)
Current smoker	16 (7.8)
Physical activity duration (min/day)**	30.0 (30.0)
Sedentary activity (hours/day)**	6.0 (4.0)
Sleep duration (hours/night)**	7.0 (2.0)
**Sleep Quality*n***, **%**	
Very good	12 (5.8)
Fairly good	94 (45.6)
Fairly bad	83 (40.3)
Very bad	17 (8.3)

**Median (IQR)

**Abbreviations**: BMI, Body Mass Index; MRS, Menopausal Rating Scale; MedDiet, Mediterranean diet; MEDAS, Mediterranean Diet Adherence Screener; SF-36, Short-form Health Survey

Overall, participants reported low-moderate adherence to a Mediterranean-style diet (5.2 ± 1.8; range: 1–11). Few participants reported achieving normative serve size criteria for core dietary components of the MEDAS including vegetables (*n* = 72 participants, 34.8%), legumes (*n* = 17 participants, 8.2%), fruit (*n* = 18 participants, 8.7%), nuts (*n* = 67, 32.4%), fish and seafood (*n* = 16 participants, 7.7%) and olive oil (*n* = 8, 3.8%). Nevertheless, almost three-quarters of participants (*n* = 148, 71.5%) reported using olive oil as their main source of dietary oil, and over three-quarters of participants reported a low frequency of sugar sweetened beverage consumption (*n* = 157, 75.8%) (Table 2).

**Table 2:** Proportion of participants achieving normative criteria for each individual MEDAS question

MedDiet food components	Achieved *n*, %
**Olive oil as main fat source**	148 (71.5)
**Olive oil serves**	8 (3.8)
**Vegetable serves**	72 (34.8)
**Fruit serves**	18 (8.7)
**Infrequent red and processed meat**	61 (29.5)
**Infrequent butter**,** margarine**,** cream**	71 (34.3)
**Infrequent sugar sweetened beverages**	157 (75.8)
**Wine**	6 (2.9)
**Legumes**	17 (8.2)
**Fish and seafood**	16 (7.7)
**Infrequent pastry and commercial sweets**	127 (61.4)
**Nuts**	67 (32.4)
**Preference for white meat over red meat**	134 (64.7)
**Tomato-based dishes**	129 (62.3)

**Abbreviations**: MedDiet, Mediterranean diet; MEDAS, Mediterranean diet adherence screener

Table 3 displays standardized beta coefficients (and 95% confidence intervals) from univariable and multivariable linear regression analysis for independent associations between adherence to a Mediterranean-style diet and severity of menopausal symptoms. Our analysis showed that adherence to a Mediterranean-style diet was not associated with severity of menopausal symptoms across each of the five regression models. When we assessed individual dietary elements included in the MEDAS, we showed that a low consumption of sugar-sweetened beverages (< 250 ml per day) was inversely associated with joint and muscle complaints, independent of age, BMI, smoking status, use of menopausal and mood medications and physical activity status (β = -0.149; CI: -0.118, -0.022; *P* = 0.042). No other significant findings for any other individual dietary element included in the MEDAS were observed.

**Table 3:** Univariable and multivariable linear regression coefficients (and 95% CI) expressing independent associations between adherence to a Mediterranean-style diet and severity of menopausal symptoms (standardised β-coefficients)**

Model	Beta (95% CI)	*P*	
1a	-0.099 (-0.060, 0.010)	0.160	
2b	-0.082 (-0.570, 0.015)	0.255	
3c	-0.077 (-0.056, 0.0170)	0.295	
4d	-0.090 (-0.061, 0.015)	0.299	
5e	-0.090 (-0.061, 0.015)	0.230	

**Standardised beta coefficient represents the change in a SD-unit increase in the Mediterranean Diet Adherence Screener score per change in outcome measure

**Abbreviations**: Beta, Standard beta coefficient

^a^Non- adjusted model; ^b^Adjusted for age and BMI; ^c^Adjusted for age, BMI, and smoking status; ^d^Adjusted for age, BMI, smoking status, use of menopause and mood medications; ^e^Adjusted for age, BMI, smoking status, use of menopause and mood medications and physical activity (mins/day)

Furthermore, adherence to a Mediterranean-style diet was positively associated with the physical function subscale of HRQoL (β = 0.173, CI: 0.001, 0.029; *P* = 0.031), independent of all covariates used in the fully adjusted model. No other subscales of HRQoL were associated with adherence to a Mediterranean-style diet. However, when assessing individual dietary constituents included in the MEDAS, our analysis showed that legume consumption (≥ 3 serves per week) was positively associated with the physical function subscale (β = 0.164, CI: 0.001, 0.004; *P* = 0.04) and a low intake of red and processed meat (≤ 1 serve per day) was positively associated with the general health subscale (β = 0.296, CI: 0.005, 0.014; *P* = < 0.001), independent of all covariates used in the fully adjusted model.

## Discussion

The primary aim of this study was to explore the association between adherence to a Mediterranean-style diet and severity of menopausal symptoms in perimenopausal and menopausal women living in Australia. We showed that adherence to a Mediterranean-style diet was not related to the severity of menopausal symptoms (as assessed by MRS). We also observed that a low consumption of sugar-sweetened beverages (< 250 ml per day), as defined by the MEDAS, was inversely associated with joint and muscle complaints, independent of all covariates. Nevertheless, these findings should be treated with caution given the potential for selection bias in the recruitment of participants and reverse causality.

Few studies have reported on the relationship between MedDiet adherence and severity of menopausal symptoms. Findings from the present study are inconsistent with previous literature suggesting that greater adherence to a MedDiet is inversely associated with the severity of menopausal symptoms [9, 22, 23]. Unlike our findings, Vetrani et al. [22] showed that the severity of menopausal symptoms, as assessed by the MRS, was inversely associated with adherence to a MedDiet in *n* = 100 post-menopausal Italian women. Furthermore, the investigators also reported that post-menopausal women with higher MRS scores had significantly lower MedDiet adherence scores compared to women with mild or no symptoms, suggesting that the severity of symptoms might be related to diet quality. Unlike the aforementioned study, our analysis controlled for important covariates including BMI, smoking status and the use of menopausal and mood medications. Moreover, Vetrani et al. [22] reported greater MedDiet adherence scores (7.8 ± 1.6 vs. 5.2 ± 1.8) when using the MEDAS compared with the present study. As such, our null findings may be due to a lack of high adherence scores reflecting the difficulties of adopting a Mediterranean-style diet in a non-Mediterranean population, making comparisons among the available literature challenging. A previous cross-sectional analysis of *n* = 606 Australian adults identified a number of important barriers towards adherence and uptake to a Mediterranean-style diet, including knowledge, motivation, affordability, time, and suitability [24]. Given that there is limited literature on the feasibility and acceptability of a Mediterranean-style dietary intervention in peri- and post-menopausal women in Australia, it is unknown whether there are unique barriers that would impede uptake and adherence within this population of women. Nevertheless, our group has previously reviewed the literature on the efficacy and adherence of a Mediterranean-style diet used as a dietary intervention in clinical trials conducted in Australia on middle-aged and older adults against primary outcomes related to cardiometabolic risk factors, glycaemic control, cognition, hepatic steatosis and depressive symptomology [25]. Although long-term adherence was possible (including sustained adherence upon completion of the dietary intervention), this was not without the implementation of intensive counselling strategies, including one-on-one support provided by Dietitians, provision of educational resources and key food items consistent with a traditional MedDiet. Moreover, there was also heterogeneity in the dietary protocols and prescriptive interpretation of a Mediterranean-style diet across all studies, with many interventions adapting a traditional MedDiet to satisfy the nutritional requirements of the population and to increase the acceptability and palatability to the multiethnic landscape of Australia [25].

Although a previous longitudinal analysis of over *n* = 6000 Australian post-menopausal women showed that adherence to a Mediterranean-style diet decreased the risk of experiencing vasomotor menopausal symptoms by 20% [9], there were important methodological differences in the way in which adherence scores were determined. Specifically, the aforementioned investigation determined a MedDiet pattern by factor analysis, using principal component analysis. A potential limitation of population-based adherence tools is that they rely on sample medians and therefore dependent on the habitual dietary characteristics of the studied population, which may limit its generalizability, particularly in non-Mediterranean populations. In the present study, we assessed MedDiet adherence using the MEDAS, which is based on normative criteria and reflective of a traditional Mediterranean-style diet. In our analysis, few participants achieved normative serve size criteria for key food groups consistent with a traditional MedDiet including olive oil (3.8%), fish and seafood (7.7%), legumes (8.2%), fruit (8.7%), nuts (32.4%) and vegetables (34.8%). Irrespective, there is also evidence from Mediterranean populations showing low to moderate adherence to a MedDiet in recent years [26]. However, it is unclear whether this reflects a true decrease in adherence given the absence of a universally accepted tool and the marked heterogeneity and psychometric properties of each of the available adherence tools [27]. The MEDAS is also not without shortcomings, particularly when applied in non-Mediterranean populations given that it was developed and validated for a specific sample population (i.e., 55–80 years at a high risk of coronary heart disease) thus potentially limiting its utility and generalizability [28].

The paradigm of assessing dietary patterns as opposed to individual nutrients or single foods as a determinant of health and wellbeing has been recognized for several decades [29]. However, few studies have investigated the relationship between diet quality and menopausal symptoms in perimenopausal or postmenopausal women. There is cross-sectional data to support that a western-style dietary pattern (e.g., high in saturated fat and sugar) is associated with severity of psychological, vasomotor, urinary-genital and somatic menopausal symptoms compared to women adhering to a plant-based dietary pattern [30, 31]. In a systematic review investigating the relationship between dietary patterns and menopausal symptoms in postmenopausal women, Noll et al. [32] reported that diets high in processed foods, saturated fats, and sugar were associated with increased psychological, vasomotor, and somatic menopausal symptoms. In the present study, we observed that a low consumption of sugar-sweetened beverages (< 250 ml per day) was inversely associated with somatic symptoms in accordance with the MRS (joint and muscle complaints). Similar findings between sugar-sweetened beverage consumption and severity of somatic symptoms have also been reported in postmenopausal women from Brazil [33]. Excessive consumption of sugar-sweetened beverages is associated with weight gain and obesity [34]. Moreover, weight gain during the menopausal transition is associated with more frequent and severe menopausal symptoms [35]. Nevertheless, in the present study, we controlled for BMI in our analysis suggesting that the severity of symptoms might be related to diet quality. Although the exact mechanisms remain unclear, excessive consumption of free sugars has been shown to disrupt lipid and energy metabolism and activate immune cells, promoting chronic inflammatory processes [34, 36] which can also intensify the severity of menopausal symptoms [37, 38].

As a secondary aim, we showed that adherence to a Mediterranean-style diet was positively associated with the physical function subscale of HRQoL. We also observed that certain dietary constituents, namely legume consumption (≥ 3 serves per week) and a low intake of red and processed meat (≤ 1 serve per day), was positively associated with the physical function and general health subscales, respectively. These findings are consistent with previous literature across a range of different cohorts, including postmenopausal women [33, 39, 40]. Given that adherence to a Mediterranean-style diet is associated with lower non-communicable disease risk [41], and that HRQoL decreases with the presence of multimorbidity [42], it is plausible that any observed relationship between adherence to a Mediterranean-style diet (or its dietary constituents) and HRQoL is secondary to a reduction in disease risk or improvements in clinical symptoms of existing disease. Nevertheless, our results may be overstated where reverse causation is plausible. Specifically, participants who report a better HRQoL may also report improved diet quality, particularly if they have received previous nutrition counselling or multi-disciplinary care. As of consequence, the associations we report may be bi-directional. Moreover, these results should be treated with caution given that it was beyond the scope of the present study to evaluate the agreement between self-perceived HRQoL and the intensity of menopausal symptoms. Therefore, the use of generic HRQoL instruments, such as the SF-36, may not identify the true health-related consequences that are unique to perimenopausal or menopausal women on individual subscales of HRQoL.

Although we observed no relationship between adherence to a Mediterranean-style diet and severity of menopausal symptoms, a previously published position statement [43] from the European Menopause and Andropause Society (EMAS) recommended that a Mediterranean-style diet should be considered in perimenopausal and postmenopausal women for attenuation of the immediate and long-term sequelae associated with postmenopausal estrogen deficiency, including vasomotor symptoms, cardiometabolic disease risk, maintenance of bone mineral density, reductions in breast cancer risk, prevention of cognitive decline and improving mood and depressive symptoms. Although further prospective studies and robust clinical trials with adequate sample sizes and clinical primary endpoints are needed, individual dietary assessment and counselling coupled with the adoption of healthy dietary patterns to mitigate the immediate and long-term health challenges associated with the female post-reproductive life stage is recommended [44].

Several limitations should be acknowledged when interpreting our study findings. Firstly, the cross-sectional study design prevents causality from being determined. Furthermore, our recruitment methods were also likely to introduce selection bias, which may limit the generalizability of our findings to the broader population of perimenopausal and post-menopausal women who were unable to participate in an online survey to ensure a more representative sample. Further to this, the lack of association between adherence to a Mediterranean-style diet and severity of menopausal symptoms may also be due to modest adherence scores, in particular a lack of participants with high adherence, again limiting the generalizability of our study findings. Another important limitation was the use of self-reported instruments to capture adherence to a Mediterranean-style diet, severity of menopausal symptoms and self-perceived HRQoL, thus increasing the potential for recall or social desirability bias. Although our multiple regression analyses were adjusted for important confounders, residual confounding cannot be ruled out. Of note, we did not consider nor assess all aspects of dietary intake (e.g., total energy intake, macro or micronutrient intake) that would typically be captured with the use of a validated food frequency questionnaire. Further to this, there is also a possibility that our multiple regression analyses were over-adjusted adding to the potential for selection bias given that some covariates used (e.g., BMI) may be considered on the causal pathway between dietary intake and severity of menopausal symptoms, thus reducing the precision of the effect estimates (e.g., influence of dietary adherence) [45].

## Conclusion

We showed that adherence to a Mediterranean-style diet was not related to the severity of menopausal symptoms in perimenopausal and menopausal Australian women. We also showed that a low consumption of sugar-sweetened beverages was inversely associated with joint and muscle complaints. Importantly, evidence on the relationship between diet quality and the intensity of menopausal symptoms is scarce. As such, our results provide an important contribution to the existing evidence on the importance of diet quality during the menopausal transition. Nevertheless, exploration of these findings using well controlled longitudinal analyses and robust clinical trials are needed to better elucidate these findings.

## Data Availability

Data that support these findings is available from the corresponding author upon reasonable request.
